# Long-Short Term Memory Network-Based Monitoring Data Anomaly Detection of a Long-Span Suspension Bridge

**DOI:** 10.3390/s22166045

**Published:** 2022-08-12

**Authors:** Jianliang Zhang, Jian Zhang, Zhishen Wu

**Affiliations:** 1 School of Civil Engineering, Southeast University, Nanjing 211189, China; 2 Jiangsu Key Laboratory of Engineering Mechanics, Southeast University, Nanjing 211189, China; 3 Key Laboratory C&PC Structures, Ministry of Education, Southeast University, Nanjing 210096, China

**Keywords:** long-span bridge, SHM system, anomaly detection, LSTM network, double thresholds

## Abstract

Structural health monitoring (SHM) systems have been widely applied in long-span bridges and a large amount of SHM data is continually collected. The harsh environment of sensors installed at structures causes multiple types of anomalies such as outlier, minor, missing, trend, drift, and break in the SHM data, which seriously hinders the further analysis of SHM data. In order to achieve anomaly detection from a large amount of SHM data, this paper proposes a long-short term memory (LSTM) network-based anomaly detection method. Firstly, the proposed method reduces the workload for preparing training sets. Secondly, the purpose of real-time anomaly detection can be met. Thirdly, the problem of high alarm rate can be avoided by utilizing double thresholds. To validate the effectiveness of the proposed method, a case study of finite element model simulation is firstly introduced, which illustrates the detailed implementation process. Finally, acceleration data from the SHM system of a long-span suspension bridge located in Jiangyin, China is employed. The results show that the proposed method can detect anomaly with high accuracy and identify abnormal accidents such as a ship collision quickly.

## 1. Introduction

SHM systems can collect structural responses which play an important role in evaluating the health condition and monitoring the exceptional events at any time, such as in ship-bridge collisions. Thus, more and more complex health monitoring systems have been installed on large-scale or critical civil structures [1,2,3,4]. In addition, many new detection methods have also been applied to bridge structures [5,6].

Meanwhile, massive SHM data is continually being collected and big data analytics is currently a hot research area. Generally, big data analysis is mainly divided into five steps which include data extraction, data storage, data cleaning, data mining, and data visualization. Among them, data mining is most widely studied, particularly for structural damage detection based on vibration and deformation data [7,8,9,10]. Unfortunately, most SHM systems work in complicated and harsh environments, which inevitably results in various abnormal data. According to the official documents, 496 sensors installed in Sutong Bridge collect 11,904 data files every day, and only about 64% data files are in good condition. The remainder of the data files are obviously abnormal in time domain or even missing, which will have a serious impact on data mining and structural health evaluation. Overall, anomaly detection is an important prerequisite for data analysis and the main purpose of this paper is to find a way to detect the anomalous SHM data with high accuracy and in real-time.

The simplest anomaly detection method is to set a static threshold for SHM data. However, detection results deeply depend on professional knowledge. Nevertheless, most traditional machine learning-based anomaly detection methods (such as Cluster, PCA, SVM, etc.) require enough data which cannot be processed in real time, so these methods cannot be applied to real-time processing time series [11,12,13,14,15,16,17].

Recently, deep learning-based methods have increasingly attracted researchers’ attention, which have been applied to all aspects. Kramer [18] utilized an autoregressive neural network to reduce noise and detect anomalies. Tamilselvan and Wang [19] utilized deep belief networks to diagnose failures in aircraft engines and electric power transformers. Abdeljaber et al. [20] utilize one-dimensional convolutional neural networks to detect structural damage. Cha et al. [21] utilized convolutional neural networks to detect crack damage detection. Allen et al. [22] utilized recurrent neural network to automatically detect pixel-level pavement cracks. Some other applications can be found in references [23,24,25,26,27,28].

Studies on anomaly detection based on deep learning have also made some progress in this field. Kambayashi et al. [29] utilized feed-forward neural networks to reconstruct the original signals; the network is trained by minimizing reconstruction errors. The well-trained network will produce larger reconstruction error with abnormal data input. Kim et al. [30] divides the data into several parts, each of which is reconstructed by deep neural network-based encoder decoder scheme; the part that has larger reconstruction error is identified as abnormal data. Filonov et al. [31] and Tuor et al. [32] utilized the prediction error of recurrent neural network (RNN) to detect the abnormal data successfully. Bao et al. [33] converted SHM data into images, and a depth-restricted Boltzmann machine (RBM) was utilized to identify the abnormal data. Ni et al. [34] used a one-dimensional convolutional neural network to extract the features of input sequences, and the fully connected network was used to recognize the abnormal data. Nanduri et al. [35] uses LSTM and GRU network to predict aircraft’s flight data, and the abnormal data are judged by a given threshold. The anomaly detection methods mentioned above which utilize neural network to reconstruct original data belongs to the offline mode and cannot provide warnings in real-time for abnormal events. Although real-time warnings can be achieved by utilizing the RNN prediction error, the RNN network itself is prone to “gradient disappearance” in the training process. The anomaly detection method which transforms data into an image actually is a kind of anomaly judgment based on computer vision, and a lot of information can be easily lost during the transformation process. The anomaly detection method which uses LSTM and GRU can process monitoring data in real-time, but the threshold given by experience does not make sense.

To solve the problems mentioned above, this paper proposes a LSTM network-based anomaly detection method, which includes two main steps: (1) an anomaly detection model including input layer, LSTM cycle module, and output layer is built; and (2) judgment criteria of double thresholds corresponding to the detection model is determined. The proposed anomaly detection method only utilizes a small amount of historical normal data in the training process, which is beneficial for SHM data with a large proportion of normal data and greatly reduces the workload of selecting normal historical SHM data in the early stage. In addition, a double thresholds method was proposed to avoid the problem of high alarm rate in the past, and the continuous input character of LSTM network can realize the aim of developing real-time warnings for abnormal events.

This paper is organized as follows. Section 2 introduces the details on the overall framework of the proposed method. Section 3 verifies the feasibility of the proposed method through a case study of finite element model simulation. In Section 4, the effectiveness of the proposed method is experimentally validated by using one day SHM data of a long-span suspension bridge when the accident of ship-bridge collision occurred. Section 5 includes the conclusion with major findings, and suggestions for further work are discussed.

## 2. Framework of the Proposed Method

**Figure 1:**
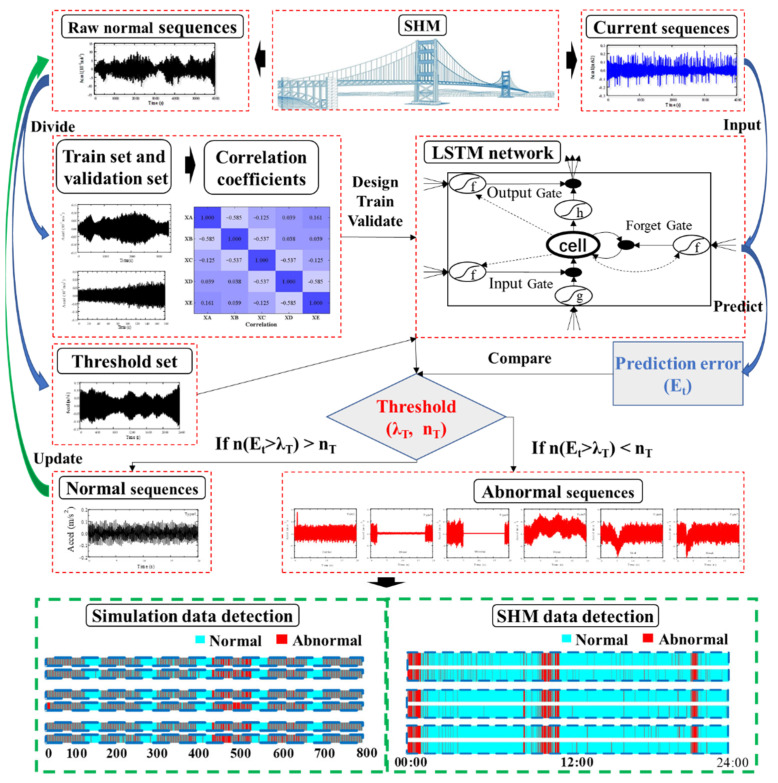
The framework of the proposed method.

The overall framework of the proposed method in this paper for detecting abnormal SHM data is shown in Figure 1. The proposed method mainly consists of four steps: data preprocessing, network prediction, thresholds decision, and abnormal detection. In the section of data preprocessing, the normal data is firstly selected from historical monitoring data manually, and then the normal monitoring data is divided randomly into three groups, named training sets, validation sets, and threshold sets. The other three steps are detailed as follows.

### 2.1. Network Prediction

The RNN is a neural sequence model that achieves state of the art performance on many important tasks. Most importantly, the RNN models allow us to operate over sequences of vectors which just meet the continuously generation characteristic of SHM data, while the convolutional neural networks (CNN) usually only accepts a fixed-sized vector as input. However, the traditional RNN usually can’t update the neuron parameters since the input sequence in the actual training process is too long [36,37]. LSTM networks differ from standard RNN networks primarily with respect to the use of memory blocks instead of neurons [38], which can solve the problem wherein traditional RNN cannot be trained when the input sequence becomes longer. Therefore, the LSTM network is selected to predict normal monitoring data in this paper.

The monitoring data recorded by sensors is composed of structural response data and random noise, and the structural response data of different channels have different data structures. This will increase the complexity of network and the difficulty of training when the monitoring data of all channels is simulated by a general network. To overcome the above problems, this paper suggests that it is necessary to establish different ‘small’ networks for each channel separately. This can avoid the problem of performance difference of a general network caused by unbalanced dataset.

In practice projects, the positions of many sensors are close (for example, they are installed on the same sling), and they can share a general network since their data structures are similar (In other words, they are highly relevant). Inspired by the covariance-driven random subspace method, the correlation coefficient is utilized to measure the correlation of monitoring data between different channels, and a threshold of correlation coefficient is necessary to determine the number of networks built. The correlation coefficient between two channels can be calculated by Equation (1):

(1) $$r_{XY}=\frac{\sum \left(X_{i}-\bar{X})(Y_{i}-\bar{Y}\right)}{\sqrt{\sum {\left(X_{i}-\bar{X}\right)}^{2}}\sqrt{\sum {\left(Y_{i}-\bar{Y}\right)}^{2}}}$$

where r is correlation coefficient, $X_{i}$ and $Y_{i}$ are monitoring data of two channels at time point i, $\bar{X}$ and $\bar{Y}$ are mean values of monitoring data.

**Figure 2:**
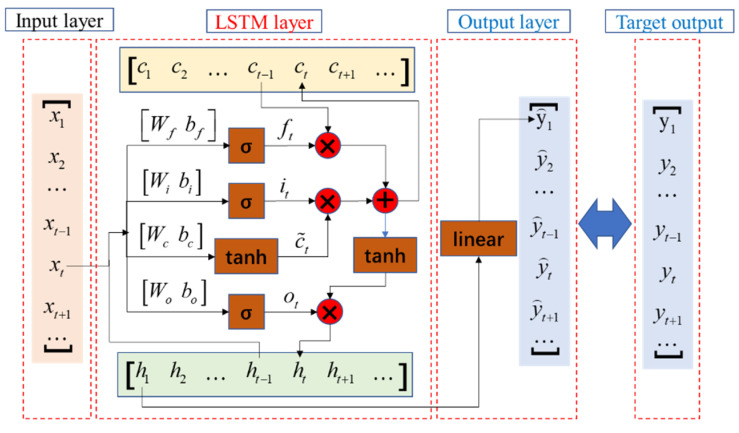
The architecture of long short-term memory (LSTM) network.

The LSTM network utilized in this paper consists of three parts: the input layer, the LSTM cycle module and the output layer (fully connected layer), as shown in Figure 2. The sets of input sequences, cell states, hidden states, output sequences and target sequences in the LSTM network are denoted as $X=(x_{1},\cdots ,x_{t},\cdots )$, $C=(c_{1},\cdots ,c_{t},\cdots )$, $H=(h_{1},\cdots ,h_{t},\cdots )$, $\overset{⌢}{Y}=({\overset{⌢}{y}}_{1},\cdots ,{\overset{⌢}{y}}_{t},\cdots )$ and $Y=(y_{1},\cdots ,y_{t},\cdots )$ separately. The cell state $c_{t}$ and hidden state $h_{t}$ can be updated by Equation (2), and the output sequence ${\overset{⌢}{y}}_{t}$ can be calculated by Equation (3):

(2) $$f_{t}=\sigma (W_{f}*[h_{t-1},x_{t}]+b_{f})\;i_{t}=\sigma (W_{i}*[h_{t-1},x_{t}]+b_{i})\;o_{t}=\sigma (W_{o}*[h_{t-1},x_{t}]+b_{o})\;c_{t}=f_{t}*c_{t-1}+i_{t}*\tanh(W_{c}*[h_{t-1},x_{t}]+b_{c})\;h_{t}=o_{t}*\tanh(c_{t})$$

(3) $${\overset{⌢}{y}}_{t}=W_{h}h_{t}+b_{h}$$

where $f_{t}$, $i_{t}$ and $o_{t}$ are forget gate, input gate and output gate in LSTM structure respectively, and the weights attached to them are $\left(W_{f},b_{f}\right)$, $\left(W_{i},b_{i}\right)$ and $\left(W_{o},b_{o}\right)$. σ denotes the sigmoid function.

The prediction process of LSTM network can be abbreviated as: ${\overset{⌢}{y}}_{t}=LSTM(x_{t},\Theta )$, where Θ denotes the trainable parameters. The training process of LSTM networks is equivalent to finding a set of training parameters to satisfy:

(4) $$\Theta_{T}=\arg\max_{\Theta }P(\left.{\overset{⌢}{y}}_{t}\right|x_{t},\Theta )$$

During the training process of LSTM network, the mean square error on training sets and validation sets are expressed as $J_{t}$ and $J_{v}$ separately, If $J_{t}$ was greater than $J_{v}$, and both $J_{t}$ and $J_{v}$ tend to be stable with increase of the number of iterations (epochs), the training processes are usually considered as well convergence. the mean square error can be calculated by Equation (5):

(5) $$J=\sum_{t}(y_{t}-{\overset{⌢}{y}}_{t}{)}^{2}$$

### 2.2. Dual Thresholds

**Figure 3:**
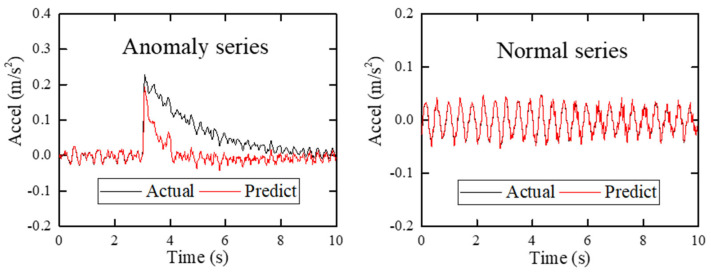
The prediction result of the LSTM network.

As mentioned earlier, for all of the training sets, validation sets and threshold sets are picked out from historical normal SHM data. The well-trained LSTM network will then perform good prediction ability with normal target sequences and poor prediction ability with abnormal target sequences, as shown in Figure 3. Therefore, the prediction error of well-trained LSTM network can reflect the anomalous possibility of target sequences, and the prediction error of each sampling point in target sequences can be calculated by Equation (6):

(6) $$E_{i}=\left|y_{i}-{\overset{⌢}{y}}_{i}\right|$$

where $E_{i}$, $y_{i}$, and ${\overset{⌢}{y}}_{i}$ are the prediction error, target value, and prediction value of sampling point i.

In order to avoid the problem of high alarm rate caused by the traditional point by point comparison of prediction error and threshold, this paper proposes a dual thresholds strategy to detect abnormal monitoring data. In the two-threshold strategy, the length of the target sequence needs to be determined firstly, which can be set according to actual requirements. Secondly, the first threshold is determined and it is compared by the prediction error of each sampling point in target sequence. Thirdly, the second threshold is determined, and it is compared by the number of sampling points in target sequence that prediction error exceeds the first threshold. Finally, the target sequence can be considered normal when the number of sampling points in target sequence where the prediction error exceeds the first threshold is less than the second threshold. Otherwise, it is considered abnormal. The above identification process of normal target sequence can be expressed briefly by Equation (7):

(7) $$n(E_{i}>\lambda_{T})<n_{T}$$

where $\lambda_{T}$ is the first threshold and $n_{T}$ is the second threshold. $n(E_{t}>\lambda_{T})$ denotes the number of sampling points wherein the prediction error exceeds the first threshold.

The probability of the occurrence event is the largest among all possible events according to the maximum likelihood estimation, so this paper reversely solves the two thresholds by maximizing the threshold set without occurrence of anomalies (or minimizing the occurrence of anomalies in threshold set). The anomalous probability of threshold sets can be obtained via statistics analysis. For generalization performance of two thresholds, it is important to avoid using training sets and validation sets instead of threshold sets, and which is selected from historical normal SHM data. To facilitate the expression of the following formulas, the number and length of target sequences in threshold sets are recorded as N and L, respectively.

First of all, all input sequences in threshold sets are fed into well-trained LSTM network, and the prediction error of all sampling points in each target sequences can be obtained. Then, the prediction errors of each target sequence are fitted by normal distribution, which is often used to simulate monitoring data The parameters of normal distribution can be gained by utilizing maximum likelihood estimation (MLE) method. According to the probability distribution equation of normal distribution, the probability that the prediction error at sampling point i is larger than the first threshold can be expressed as:

(8) $$P_{i}(E_{i}>\lambda_{T}\left|(u,\Sigma \right.))=2(1-\Phi (\frac{\lambda_{T}-u}{\Sigma }))$$

where $P_{i}$ denotes the probability of anomaly occurring at sampling point i. Φ(•) denotes the probability distribution equation of standard normal distribution. $\left(u,\Sigma \right)$ denotes the two parameters of normal distribution. $\lambda_{T}$ is the first threshold, and $\lambda_{T}\in [0,E_{\max}]$. $E_{\max}$ is the maximum prediction error in the threshold sets.

According to the probability of anomaly occurring at each sampling point, the anomalous probability of each target sequence can be obtained by utilizing binomial distribution analysis technique:

(9) $$P_{S}(n(E_{i}>\lambda_{T})>n_{T}\left|(u,\Sigma \right.),L)=\sum_{j=N_{T}+1}^{L}\frac{L!}{j!(L-j)!}P_{i}^{j}{\left(1-p_{i}\right)}^{L-j}$$

where $P_{S}$ denotes the anomalous probability of each target sequence. $n_{T}$ is the second threshold, and $n_{T}\in [0,L]$.

According to the anomalous probability of each target sequence, the anomalous probability of threshold sets can be expressed as:

(10) $$P_{T}(n(n(E_{i}>\lambda_{T})>n_{T})>1\left|(u,\Sigma \right.),L,N)=1-{\left(1-P_{S}\right)}^{N}\;$$

where $P_{T}$ denotes the anomalous probability of threshold sets.

Unlike the first threshold, both the second threshold and the length of target sequences are determined according to actual requirements. Since the target sequences of threshold set are collected from normal historical normal SHM data, the value of the first threshold should minimize the anomalous probability of threshold sets for any given length of target sequences and the second threshold. In other words, the value of first threshold need to satisfy the following Equation (11):

(11) $$\lambda_{T}=\arg\min_{\lambda_{T}}P_{T}\;$$

The values of the first threshold can be obtained from Equations (8)–(10):

(12) $$\lambda_{T}=E_{\max}\;$$

In order to minimize the anomalous probability of each target sequence in the threshold set, the second threshold can be obtained from Equation (12) for the first threshold determined:

(13) $$n_{T}=P_{i}(E_{i}>E_{\max})*L$$

### 2.3. Anomaly Detetction and Evaluation Criterias

After the two thresholds are determined, the abnormal data can be identified from current monitoring data. Firstly, the current monitoring data needs to be recombined into input sequences and target sequences according to network requirements. Then, the input sequences are fed into the predictive network and the prediction error of the target sequences is calculated. Finally, we can easily identify abnormal monitoring data by comparing prediction error of target sequences with dual thresholds.

In order to evaluate the performance of proposed anomaly detection method, the concepts of anomaly rate (AR), anomaly detection rate (ADR), and anomaly detection accuracy (ADA) are introduced. Anomaly rate is the ratio of the abnormal target sequences number to the total of target sequences number. Anomaly detection rate is the rate of identified abnormal target sequences number to the total of target sequences number. Anomaly detection accuracy is the ratio of the identified abnormal target sequences number to the actual abnormal target sequences number. In addition, the concepts of classic precision (P), recall (R), and accuracy (A) are also introduced. The detection results of target sequences are only included normal and abnormal, which belongs to the two-classification problem. The abnormal sequences are set as positive, and the normal sequences are set as negative. TP means the number of abnormal sequences identified as positive, FP means the number abnormal sequences identified as negative, FN means the number of normal sequences identified as positive, and TN means the number normal sequences identified as negative. AR, ADR, P, R, and A can be calculated as follows:

(14) $$\mathrm{AR}=\frac{\mathrm{TP}+\mathrm{FP}}{\mathrm{TP}+\mathrm{TN}+\mathrm{FP}+\mathrm{FN}}\;\mathrm{ADR}=\frac{\mathrm{TP}+\mathrm{FN}}{\mathrm{TP}+\mathrm{FP}+\mathrm{FP}+\mathrm{FN}}\;\mathrm{ADA}=\frac{\mathrm{TP}+\mathrm{FN}}{\mathrm{TP}+\mathrm{FP}}\;P=\frac{\mathrm{TP}}{\mathrm{TP}+\mathrm{FP}}\;R=\frac{\mathrm{TP}}{\mathrm{TP}+\mathrm{FN}}\;A=\frac{\mathrm{TP}+\mathrm{TN}}{\mathrm{TP}+\mathrm{TN}+\mathrm{FP}+\mathrm{FN}}$$

## 3. Case Study 1: Numerical Example

### 3.1. Structural Response Simulation and Data Preprocessing

To validate the effectiveness of the proposed method, a case study of finite element model simulation is firstly introduced. Compared with the SHM data of real structures, varies types of anomalies can be easily added to finite element model response which will benefit for the accurate human labeled. A simply supported H-beam with length of 1.2 m is considered in this study. The cross-sectional area and inertial moment of the beam are 24.37 cm^2^ and 999.97 cm^4^, respectively. The 1/6 span, 2/6 span, 3/6 span, 4/6 span, and 5/6 span of the beam are simultaneously excited by white Gaussian noise. At the same time, the structure response of these excitation points is collected with 50 Hz sampling frequency, and five normal structural responses with 500,000 sampling points are obtained. In order to increase the difficulty of anomaly detection, noise with 20 signal-to-noise ratios (SNR) is added to these responses, and these noise-added responses are named as XA, XB, XC, XD, and XE, respectively.

In order to better illustrate the effectiveness of the anomaly detection method proposed in this paper, the five simulated responses need to be spilt into training sets, validation sets, threshold sets, and test sets. Before the sets are established, five simulated responses are continuously intercepted into the input sequences by a time window of 270 sampling points with an interval of 250 sampling points. Taking response XA as an example, the number of input sequences is 1999. These input sequences are randomly assigned to four sets, and the number of input sequences in training sets, validation sets, threshold sets and test sets are 760, 39, 400, and 800 separately. The target sequence corresponding to each input sequence consists of the last 250 sampling points of input sequences.

**Figure 4:**
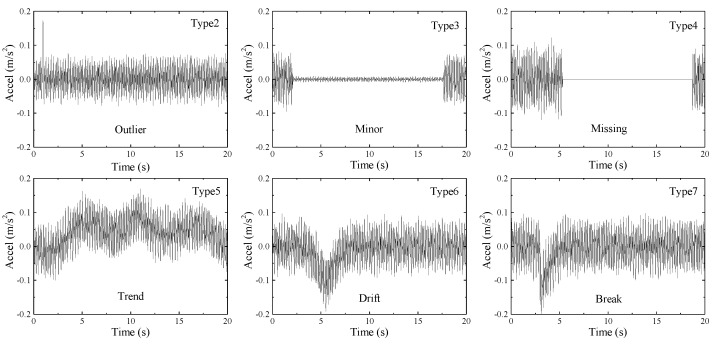
Six typical anomaly patterns of acceleration data.

In order to verify the universality of the proposed anomaly detection method, six kinds of abnormal patterns are designed for each test set, which are outlier (Type 2), minor (Type 3), missing (Type 4), trend (Type 5), drift (Type 6), and break (Type 7), respectively, as shown in Figure 4. The method of building abnormal input sequences and corresponding abnormal target sequence is to replace normal data with abnormal data. As a result of each test set, the abnormal pattern of input sequences 1–100 is type 2, 141–240 is type 3, 281–380 is type 4, 421–520 is type 5, 561–660 is type 6, 701–800 is type 7. The unchanged input sequences are labeled as normal (Type1).

### 3.2. Network Training and Two Thresholds Determination

**Figure 5:**
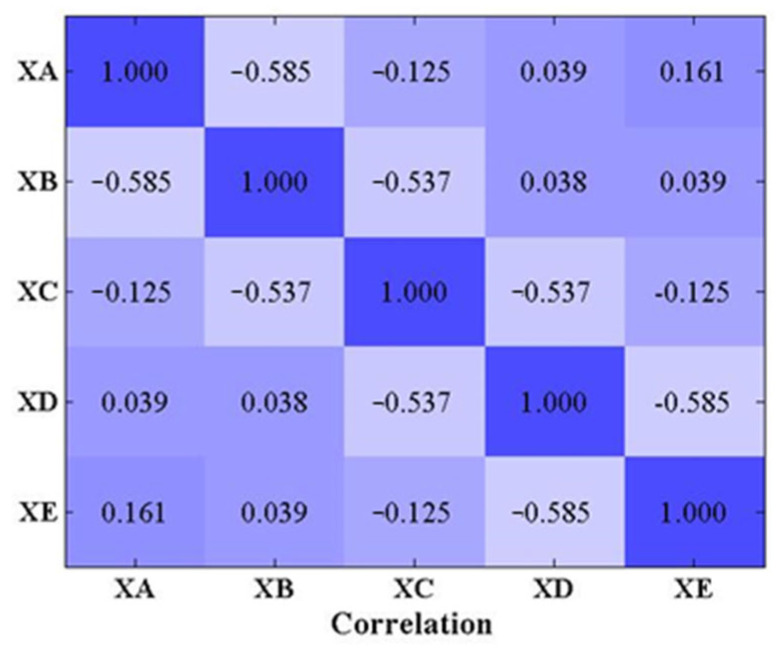
The correlation coefficients between original signals.

To determine the number of LSTM networks established, the correlation coefficients of abnormal input sequences in the five test sets are shown in Figure 5. Since the noise ratio of the response is 20%, the threshold for the correlation coefficient is set at 80%. According to the calculation results in Figure 5, five LSTM networks need to be established to predict the above five response data, and they are named Net1, Net2, Net3, Net4, and Net5.

**Figure 6:**
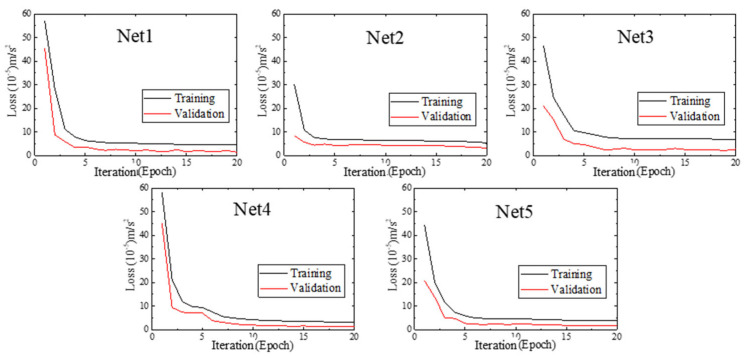
The training processes of built networks.

During the training processes, the basic parameters of the five designed networks are set as follows: the input and output dimensions of the networks are 270 and 250, respectively, the batch size is 256, dropout is 0.3, and epoch is 20. The activation function of the fully connection layer is linear function, the loss function is the mean square error (MSE). These networks are trained using Adam optimizer. The mean square errors on training sets and validation sets of each network are shown in Figure 6. It can be seen that all of these networks converge gradually with increasing of iterations (epochs).

To obtain two thresholds, the input sequences of threshold sets are fed into the well-trained LSTM networks to get the prediction errors. In order to increase the stability of the detection results, the prediction errors of each input sequence are averaged by a five-sampling-point sliding window with a stride of one sampling point. Then the first threshold can be calculated by Equation (15), and the results corresponding to Net1-Net5 are shown in Table 1. According to Equation (13), the value of second thresholds corresponding to Net1–Net5 are all calculated as one.

(15) $$\begin{array}{l} \lambda_{s}=\max((E_{i}+E_{i+1}+E_{i+2}+E_{i+3}+E_{i+4})/5)\;i\in [1,246]\\ \lambda_{T}=\max(\lambda_{s})\;s\in [1,400] \end{array}$$

where $\lambda_{s}$ is the maximum prediction error of each target sequence.

### 3.3. Results and Analysis

After the networks are trained and the two thresholds are determined, the anomaly data in the target sequences of test sets can be identified. Similarly, the prediction errors need to be averaged by a five-sampling-point sliding window with a stride of five sampling point. The anomaly detection results are shown in Table 2. From Table 2, we can find that the detection results are essentially in agreement with the correlation coefficients in Figure 5, In other words, the number of trained LSTM networks must be equal to the number of channels for multi-channel data with weak correlation.

**Figure 7:**
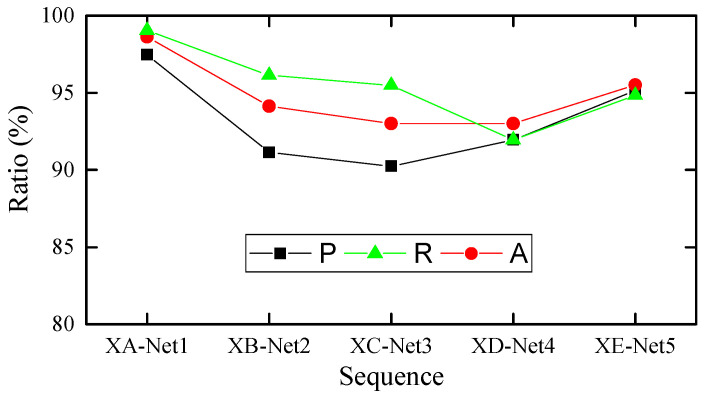
The precise (P), recall (R) and accuracy (A) of the detection results.

**Figure 8:**
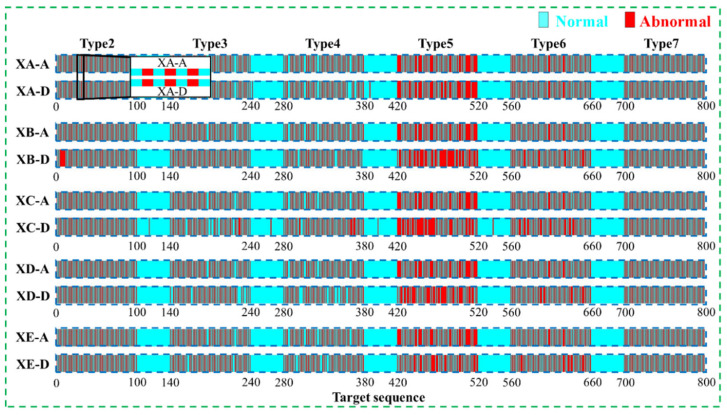
The detection results of numerical example (-A symbolizes the results with human labeled, -D symbolizes the detected results).

In order to further illustrate the effectiveness of the proposed anomaly detection method in identifying various abnormal patterns, Table 3 shows the detection results of six abnormal patterns in each test set. From the Table 3, it can be seen that the proposed method can achieve good detection results for six abnormal patterns. Precision, recall, and accuracy are calculated to evaluate the overall performance of the proposed method in each test set, as shown in Figure 7, and the actual anomaly layout and the detection results are compared in Figure 8.

## 4. Case Study 2: Experimental Example of a Long Span Suspension Bridge

### 4.1. Background

**Figure 9:**
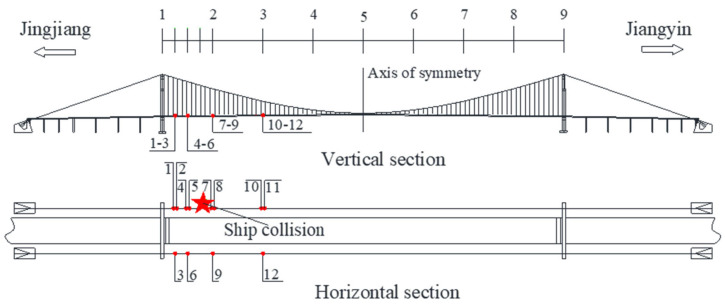
The layout of accelerometers installed in suspenders.

**Figure 10:**
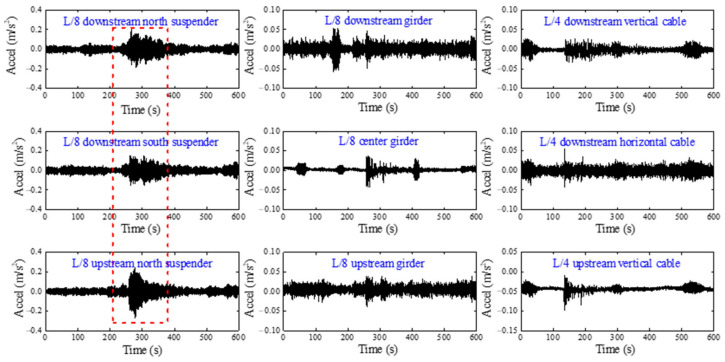
The acceleration data under ship collision of Jiangyin Bridge.

To further illustrate the extensive applicability of the proposed method, the acceleration data of a long-span suspension bridge located in Jiangyin, China is employed. According to historical official records, the Jiangyin Bridge was hit by a ship in 2 June 2005, and the impact location on the main girder of the bridge is shown in Figure 9. Figure 10 shows the typical response data of the accelerometers on the suspender, girder and main cable which are closest to the ship impact position, respectively. As can be seen from Figure 10, the acceleration data of the suspenders is most sensitive to ship impact accidents. Therefore, the accident of ship collision can be easily identified based on suspenders’ acceleration data. A total of 12 accelerometers are installed on the suspenders of Jiangyin Bridge, as shown in Figure 9.

### 4.2. Data Preprocessing

**Figure 11:**
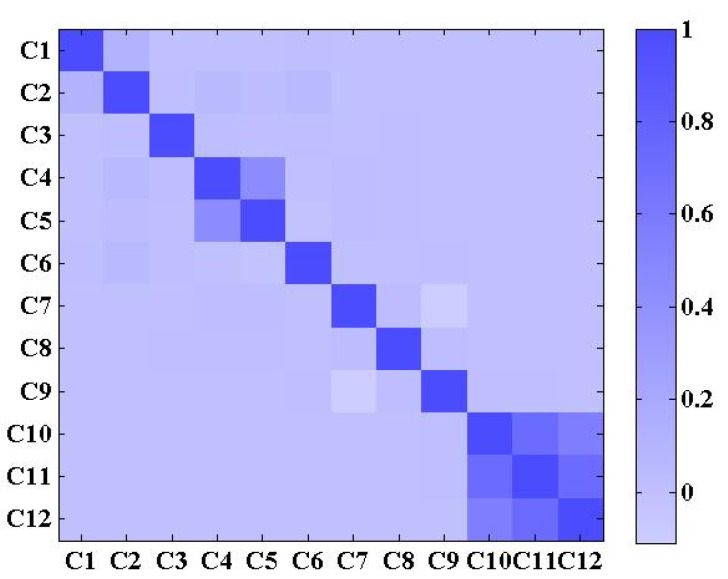
The correlation coefficients between different channels’ monitoring data.

In this section, the suspender acceleration data from 1 June to 2 June 2005 is employed. The calculated correlation coefficients of 12 channels are shown in Figure 11, and the maximum correlation coefficient of different channels is 0.729. The threshold for the correlation coefficient should be determined according to the proportion of noise in the response data. Here, 80% is adopted as a rule of thumb. Therefore, it is necessary to train 12 different LSTM networks to correspondingly detect anomaly in 12 channels, and these networks are named as LSTM1~LSTM12, respectively. To create training sets, validation sets, threshold sets, and test sets, the acceleration data of 12 channels is continuously intercepted into the input sequences by a time window of 1050 sampling points with an interval of 1000 sampling points. The training sets, validation sets, and threshold sets were randomly selected from the normal input sequences on 1 June 2005. For each channel, the number of input sequences in training sets, validation sets, and threshold sets are 342, 18 and 36 respectively. All of the input sequences on 2 June 2005 are utilized as the test sets, and the number of input sequences is 4320. The target sequence corresponding to each input sequence consists of the last 1000 sampling points of input sequences.

### 4.3. Network Training and Dual Thresholds Determination

**Figure 12:**
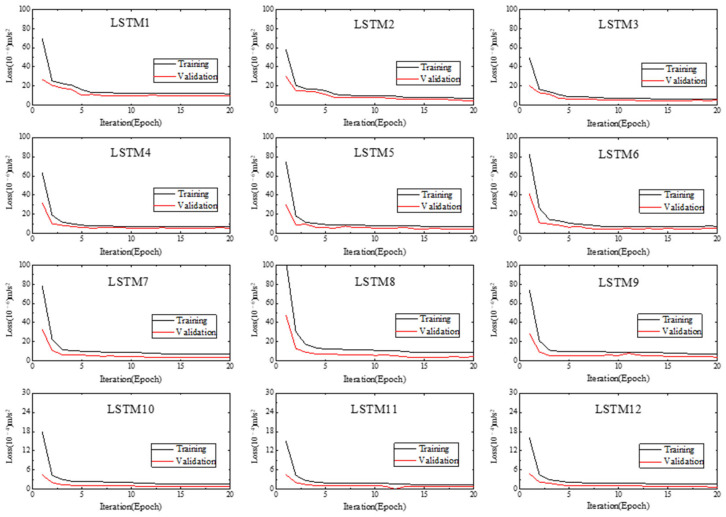
The training and validation processes.

The Adam optimization strategy is adopted to train the 12 LSTM networks. The input and output dimensions of these networks are set to 1050 and 1000 separately, and other parameters have the same setting with Section 3.2. The mean square errors on training sets and validation sets of networks are shown in Figure 12. It can be seen that the prediction errors on training sets is greater than the prediction errors on validation sets, and both of them tend to be stable with increasing of iterations (epochs), which means that the training results are pretty good.

Similar to the case study of finite element model simulation in Section 3, the prediction error are firstly averaged by a five-time-point window. The first thresholds corresponding to LSTM1-LSTM12 can be obtained by Equation (15), as shown in Table 4. all of the second thresholds corresponding to LSTM1-LSTM12 are calculated as 1.

### 4.4. Result and Discussion

**Figure 13:**
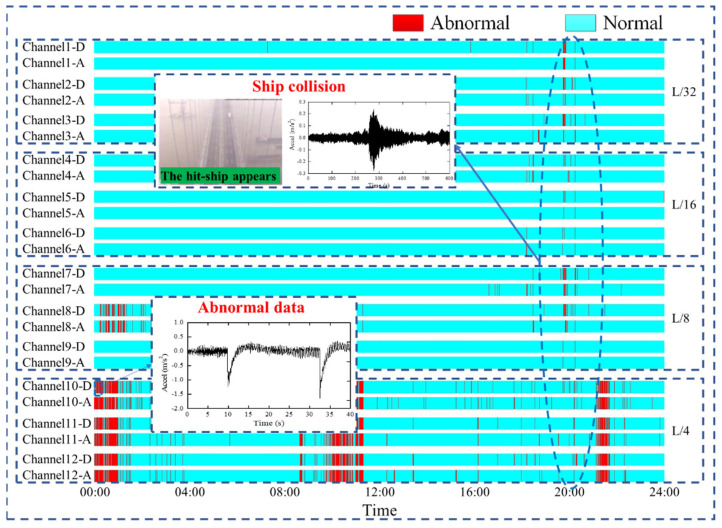
The detection results of SHM data (-A symbolizes the results with human labeled, -D symbolizes the detected results).

The human labeled results and networks detection results of 12 channels are compared in Figure 13. The ship collision accidents can be quickly located while the acceleration data of 12 channels are detected as anomaly at the same time point. The accuracy of detection results in each channel are given in Table 5, and it can be seen that the proposed method performs well in real bridge data processing and exception event warning.

**Figure 14:**
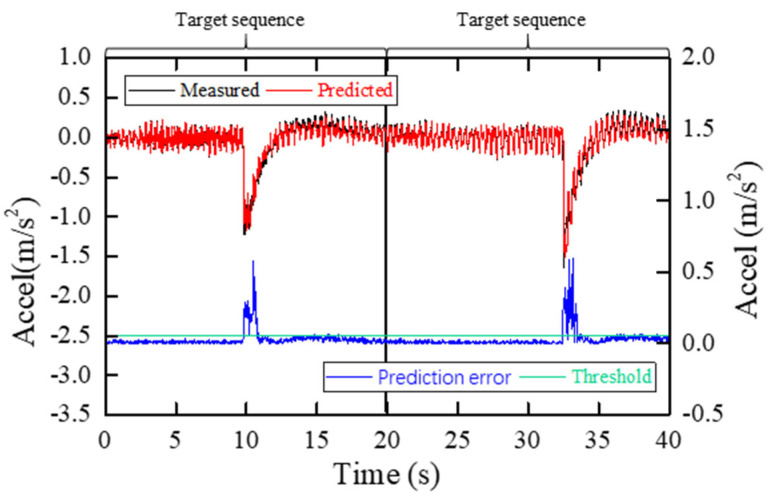
An example of anomaly detection with proposed method.

In order to better explain the mechanism of proposed method, an anomaly detection example for two target sequences in this case study is shown in Figure 14. The left ordinate is the value corresponding to the target sequences and the output sequences, and the right ordinate is the value corresponding to the prediction error and the first threshold. As can be seen from Figure 14, the prediction errors of more than 50 sampling points in both target sequences exceed the first threshold, so both target sequences are judged as anomalies. If a single threshold is used, both target sequences will alarm for more than 50 times, which is obviously unreasonable.

In order to illustrate the advantages of the anomaly identification method in this paper in workload, the data set preparation method in this paper is compared with [31]. Firstly, this paper only needs to prepare normal dataset, while [31] prepares both normal and abnormal datasets. Secondly, each channel in this paper establishes a “small” network independently, so there is no need to pay attention to the balance of data between channels, while [31] needs to pay attention to this aspect. Finally, the data structure of each channel is relatively fixed, so only a few sample data are needed to learn the “small network”.

## 5. Conclusions

The main contribution of this paper is to propose an anomaly detection method based on LSTM network. First, a small amount of historical normal data is used to train the network. Then, the prediction error of the well-trained network can be utilized to detect the anomaly. The feasibility of the method has been verified by two case studies of finite element simulation and real suspension bridge SHM data successively. Compared with other anomaly detection methods, the major and novel contributions here are as follows:

- An anomaly detection method based on LSTM network is proposed, which not only achieve the requirement of real-time identification, but also reduces the workload of preparing training sets.
- The correlation coefficient is used to determine the number of networks that need to be established. The problem of performance differences of networks in different channels caused by unbalanced dataset can be avoided by establishing the “small” concept for each channel.
- A double thresholds method was proposed to avoid the problem of high alarm rate in the past.
- The anomaly detection method is applied to the acceleration data of Jiangyin Bridge, and the ship collision accident of Jiangyin Bridge is successfully detected, which proves the application potential of this method in the structural warning system.

Despite the aforementioned advantages of the proposed method, this paper only considers the time-domain data itself. In the next step, structural parameters such as frequency, mass, and stiffness will be introduced as input of network to improve the detection effect of the anomaly.

In addition, the anomaly of SHM data are either caused by abrupt change of structure performance and external load or by the faults of sensors themselves. The former can reflect the structural characteristics and needs to be accurately captured. The latter has no practical significance and needs to be repaired. Therefore, the question concerning how to effectively distinguish the two types of anomalies will be the focus of future work.

## Figures and Tables

**Table 1:** The first threshold of Net1~Net5.

Item	Threshold				
	Net1	Net2	Net3	Net4	Net5
$\lambda_{T}(10^{-3}m/s^{2})$	10.6	11.1	16.1	14.4	9.7

**Table 2:** Anomaly detection results of test sets.

Responses	AR	ADR				
		Net1	Net2	Net3	Net4	Net5
XA	38.75%	39.38%	40.63%	39.75%	39.13%	39.00%
XB	38.75%	98.75%	40.88%	99.00%	39.13%	98.88%
XC	38.75%	98.38%	98.13%	40.63%	98.25%	98.25%
XD	38.75%	98.50%	40.63%	98.38%	38.75%	98.88%
XE	38.75%	38.63%	39.75%	39.00%	38.75%	38.63%

**Table 3:** Anomaly detection results of six types of abnormal patterns.

Abnormal Patterns	AR (Type)	ADR (Type)				
		Net1-XA	Net2-XB	Net3- XC	Net4-XD	Net5-XE
Type1	6.25%	6.38%	6.63%	6.38%	6.25%	6.25%
Type2	6.00%	6.00%	6.25%	5.88%	5.63%	6.00%
Type3	5.75%	5.63%	5.88%	6.50%	5.55%	5.88%
Type4	8.00%	8.63%	9.13%	8.75%	8.38%	7.83%
Type5	6.50%	6.50%	6.75%	7.25%	6.75%	6.88%
Type6	6.25%	6.25%	6.25%	6.25%	6.25%	6.25%

**Table 4:** The first threshold of LSTM1~LSTM12.

Model	*λ_T_* (10^−3^ m/s^2^)	Model	*λ_T_* (10^−3^ m/s^2^)	Model	*λ_T_* (10^−3^ m/s^2^)
LSTM 1	15.4	LSTM 5	18.2	LSTM 9	16.9
LSTM 2	19.8	LSTM 6	20.7	LSTM 10	52.4
LSTM 3	13.6	LSTM 7	13.3	LSTM 11	49.3
LSTM 4	16.2	LSTM 8	17.0	LSTM 12	46.7

**Table 5:** The ADA of 12 channels.

Sensors	ADA (%)	Sensors	ADA (%)	Sensors	ADA
Channel 1	99.5	Channel 5	99.7	Channel 9	99.9
Channel 2	99.5	Channel 6	99.9	Channel 10	96.9
Channel 3	99.2	Channel 7	98.8	Channel 11	97.7
Channel 4	99.3	Channel 8	99.0	Channel 12	97.4

## Data Availability

Not applicable.
